# Drivers of managed entry agreements to reduce reimbursement challenges of orphan medicinal products: the development of a matrix

**DOI:** 10.1186/s13023-025-04020-8

**Published:** 2025-10-27

**Authors:** Marcelien H. E. Callenbach, Sibren van den Berg, Alisa Hulsbosch, Carla E. M. Hollak, Christine Leopold, Aukje K. Mantel-Teeuwisse, Wim G. Goettsch

**Affiliations:** 1 Division of Pharmacoepidemiology & Clinical Pharmacology, Utrecht Institute for Pharmaceutical Sciences (UIPS), Utrecht University, Utrecht, The Netherlands; 2 Medicine for Society, Platform at Amsterdam UMC location University of Amsterdam, Amsterdam, The Netherlands; 3 Amsterdam UMC, University of Amsterdam, Endocrinology and Metabolism, Amsterdam Gastroenterology Endocrinology Metabolism, Amsterdam, The Netherlands; 4 National Health Care Institute (ZIN), Diemen, The Netherlands

## Abstract

**Background:**

Reimbursing orphan medicinal products (OMPs) presents both opportunities and challenges for national healthcare payers and health technology assessment (HTA) bodies because of their potential high benefits, large clinical uncertainties and high prices. To support a more structured application of (outcome-based) managed entry agreements (MEAs) intended to mitigate these OMP-related reimbursement challenges, a matrix was developed to facilitate reimbursement negotiations and, ultimately, patient access.

**Methods:**

A systematic literature review was performed, searching PubMed, Embase, and grey literature from 1 January 2000 until 1 January 2024 to globally identify reimbursement challenges (clinical-, cost-effectiveness uncertainties, or financial risks) described in relation to MEAs for OMPs. The data retrieved were used to develop a matrix that structures the drivers of managed access agreements to reduce financial risk and reimbursement challenges specific to OMPs.

**Results:**

A total of 77 studies were included in the review, identifying 23 different types of MEAs for OMPs. The results indicated that more commonly known MEAs were designed to mitigate different reimbursement challenges, and more innovative MEAs and combinations thereof have been frequently described in literature. The selected case study of Myozyme^®^ illustrated how the matrix can present stakeholders with additional mitigation strategies for the relevant reimbursement challenges.

**Conclusion:**

To address reimbursement challenges for OMPs along their life cycle, it is valuable to consider both established and innovative, e.g., outcome-based MEAs. Combining reimbursement and payment models has the potential to address multifaceted reimbursement challenges. The developed matrix fills a gap in providing a structure for drivers of MEAs tailored to OMPs, enhancing decision-making processes and ultimate patient access to OMPs targeting high unmet medical needs.

**Supplementary Information:**

The online version contains supplementary material available at 10.1186/s13023-025-04020-8.

## Introduction

New orphan medicinal products (OMPs) are increasingly expensive and approved with limited clinical evidence [1]. OMPs can be defined as therapies for “orphan conditions” with a prevalence of not more than five in 10,000 persons in the European Union (EU) [2]. Due to legislation incentivising orphan drug development, such as conditional authorisation or marketing under exceptional circumstances or 10 year market exclusivity, and scientific advice, currently around 50% of new marketing authorisations are for OMPs targeting unmet needs for rare conditions [3]. Nevertheless, patient access is still often hampered due to significant reimbursement challenges. One of the reasons for these challenges is the limited evidence base of OMPs due to uncontrolled or small randomised controlled trials of short duration and the absence of robust evidence on clinically relevant outcomes, which makes it more difficult to demonstrate patient benefit [4, 5]. These factors limit strong conclusions, making both the effectiveness and cost-effectiveness of these drugs uncertain at the time of a reimbursement decision. Another reason is the often substantial prices of OMPs, large upfront payments and/or high budget impacts which present financial risks to national competent authorities for pricing and reimbursement (NCAPRs). This introduces the risk of not only allocative inefficacy when resources are not used where they create the most value, but also technical inefficacy when resources are not used in the best way to produce the most output [6, 7]. Even though these three components of reimbursement challenges, (1) clinical effectiveness uncertainties, (2) cost-effectiveness uncertainties and (3) financial risks, are not unique to OMPs, the level and combination at which they occur are more prominent [8]. Consequently, the answer to the question of whether or to what extent a patient will benefit from a new OMP and whether it provides value for money, from a healthcare payer’s perspective, is not straightforward.

To mitigate this ‘grey area’ for OMPs, where potential benefits may exist but come with high prices, NCAPRs, HTA bodies, and health technology developers (HTDs) have collaborated on managed entry agreements (MEAs) [9–14]. These arrangements can use a variety of mechanisms to address clinical uncertainty about OMPs performance or to manage reimbursement challenges, e.g., limit their budget impact. MEAs can be categorised into purely financial or outcome-based reimbursement models, in combination with payment models to arrange if the payment will be made upfront or delayed (e.g., annuity payments, pay at outcome achieved), which can be executed at a (sub)population or individual patient level [9–13]. Outcome-based reimbursement and delayed payment models have received more interest among HDTs and NCAPRs, including in rare diseases, and recent research shows a growing appetite for implementing these agreements [9–11, 13–16]. Nevertheless, significant challenges exist in the operationalisation and implementation of MEAs, given the potential complexity involved with these agreements [13, 16–23]. Research has outlined the differences in MEA uptake amongst countries for similar therapies and showed that the perception of the type and level of uncertainty can be a main driver in MEA application and, ultimately, a positive/negative reimbursement outcome [24–30]. Deciding which MEA is most suitable can be delicate and time-consuming, especially for the more innovative and complex MEAs, such as outcome-based reimbursement models. Therefore, having a better understanding of suitable MEAs early in the development trajectory could support timely reimbursement decision-making in the future. Existing tools or frameworks have already been recognised for assisting stakeholders in defining uncertainties, describing MEAs based on their application in different settings and perceived barriers and providing guidance in negotiating them [29, 31–35]. Nonetheless, a systematic overview linking the type of uncertainty and financial risk to the most suitable type of MEA is currently missing. Moreover, research has shown that “one size does not fit all” and that the perception of uncertainties and risks can change over time, creating the need for a tailored approach to identify which reimbursement challenges different mitigation strategies throughout the lifecycle of therapies.

Therefore, this study aims to develop an OMP-specific matrix to link various types of MEAs (including reimbursement models, payment models, or combinations thereof) with specific clinical and cost-effectiveness uncertainties and/or financial risks that can be used at different stages of the drug lifecycle. With the developed matrix, this paper aims to support NCAPRs, HDTs and Health Technology Agencies (HTA) bodies in advising on and deciding upon appropriate MEAs, thereby mitigating reimbursement challenges inherent to OMPs and facilitating improved patient access to OMPs in a sustainable fashion.

## Methods

The matrix was developed through a systematic literature review analysing which MEAs have been described for what reimbursement challenges. A case study was selected from the extracted literature to showcase the usability of the matrix. Using the matrix, additional MEAs - other than the already reported MEAs - that could mitigate the reported reimbursement challenges for the selected case study were identified, offering stakeholders new options which may not have been considered before.

### Systematic literature review

A systematic review following PRISMA guidelines and Cochrane collaboration recommendations was conducted in PubMed, Embase and grey literature on 12 February 2024 to develop the matrix [36, 37]. The search strategy included two search strings: the first element related to orphan diseases and the second element related to MEAs, which contained a variety of synonyms (the search string can be retrieved in supplementary materials I). Studies (excluding abstracts) were included if they were published in a peer-reviewed journal, describing managed entry agreements for OMPs and what reimbursement challenges they aim to mitigate, written in English and published between 1 January 2000 and 1 January 2024. No restrictions were applied regarding the type of OMP, jurisdiction (a global approach was taken) or the country setting (higher- vs. lower-income). Using the same search strategy, targeted grey literature was hand-searched at the website of relevant organisations and projects within the HTA field (e.g. OECD, ISPOR, HTAi, WHO, Impact HTA and the Oslo Initiative).

After the search, studies were excluded using Rayyan [38] (an artificial intelligence tool) for (1) duplicate removal, (2) language exclusion (non-English) and (3) title/abstract exclusion based on a pre-defined list of keywords (listed in supplementary materials II). Title and abstract screening were not applied for grey literature because abstracts are often unavailable in grey literature papers. Executive summaries and tables of contents were used to assess article eligibility instead. The selected studies were then subject to full-text screening in Mendeley 2.85.0. Unretrievable articles were excluded. The full-text screening was done by two authors (AH and MC); if they did not mention MEAs in the area of rare diseases (in general or with the mentioning of a specific disease or product), uncertainty management or relevant synonyms, they were excluded.

During the data extraction procedure, reference lists were hand-screened to identify potentially relevant additional studies (snowballing). Similarly, manual searches were also performed by searching for relevant studies in the reference lists of included sources from grey literature.

### Data extraction

A data extraction template was developed in Excel, which included the following variables: title; publication date; author; product, therapy type or disease area; MEA name(s); description of MEA; level of MEA application (patient or (sub)population); and MEA component (reimbursement model, payment model or a combination of both); and a description of the clinical- and/or cost-effectiveness uncertainties or other reimbursement challenges for OMPs. Using this extraction template, information was extracted from the final list of included studies. Multiple reimbursement challenges could be extracted from one sentence. For bias reduction, all data inputs were examined independently by MC to crosscheck the validity of the data extracted.

### Classification of managed entry agreements and reimbursement challenges

The extracted MEAs were classified deductively and inductively. Given that there are many different taxonomies in the literature that describe and categorise MEAs, we first took a set of commonly used MEA taxonomies from previous literature, especially from our previous paper on managed entry agreements [34, 35]. In line with this taxonomy and others, we considered reimbursement models (outcome- or financial-based) and payment models (e.g., upfront payment or delayed payment). Second, the extracted MEAs were deductively classified by assessing whether the described MEA name or its description matched or was closely related to any of the predefined taxonomies. Third, their similarity was assessed for those that did not conform with the existing taxonomies, and MEAs were clustered and inductively categorised under a new MEA category. For example, outcome-based staged payments and performance-based annuities were clustered under pay-for-outcome with annuity payments. Finally, given that reimbursement and payment models are not mutually exclusive, the taxonomy that is used classified them separately, and each uniquely described combination was inductively added.

Based on the previously identified literature, the category of reimbursement challenges was further subcategorised into three components: (1) clinical effectiveness uncertainties, (2) cost-effectiveness uncertainties and (3) financial risks. Deductively, clinical effectiveness uncertainties were structured and classified using the PICOTS framework, commonly used to structure and describe basic characteristics of the disease (Population, Intervention, Comparator, Outcome(s)), Time frame, and Study Design [39, 40]. Cost-effectiveness uncertainties were subdivided into uncertainties concerning models, input parameters and output [39]. Financial risks were subdivided into risks associated with high upfront payments or budget impact. If other reimbursement challenges were extracted from literature that did not fit the initial classification, inductively, alternations were made, and new categories were included.

### Development of the OMP-specific matrix linking meas and reimbursement challenges

With the use of the extracted and categorised MEAs and reimbursement challenges for OMPs, an OMP-specific matrix was developed that structures the drivers of managed access agreements to reduce risk and reimbursement challenges specific to OMPs. The elements of MEAs were structured based on whether the included literature reported and described an MEA designed for specific reimbursement challenges (categorised as intended to mitigate reimbursement challenges (risk mitigation design) or not (categorised as no risk mitigation design if specifically mentioned as such or not mentioned).

### Case study selection

A case study was selected by first identifying which specific OMPs were described in the extracted information from the systematic literature. The authors discussed/assessed these OMPs together and, with their expertise and knowledge from past experiences, identified a product representing common OMP reimbursement challenges relevant to future healthcare decision-making. (Supplementary materials III provides more details about the selected case study).

## Results

### Literature inclusion

A total of 792 references were identified through the systematic literature search. From this list, 768 references were identified from the two selected databases and 24 from grey literature. The PRISMA flowchart can be found in supplementary materials IV. After the removal of duplicate references and references not written in English, a total of 650 references were subject to title and abstract screening for references from the databases and executive summaries and tables of contents screening for the grey literature. References that had no full-text (publicly) available or were out of scope based on the inclusion-exclusion criteria were excluded. Using backwards snowballing, a second reference search was conducted by hand-searching grey literature and relevant references from the retrieved articles, resulting in 37 additional references. This led to a total of 223 references that were selected for full-text review. Applying the final exclusion criteria resulted in 77 remaining articles for the data extraction, which were included in the development of the OMP-specific matrix.

### Matrix that structures the drivers of managed access agreements for omps

From the 77 papers describing specific MEAs designed for OMP-related reimbursement challenges, 23 distinct MEAs were identified in literature (taxonomy in supplementary materials V). Four were financial-based reimbursement models, three outcome-based reimbursement models, four payment models, and 12 combined models. Table 1 presents a graphical overview of the potential suitability of the extracted MEAs for the different reimbursement challenges for OMPs (in supplementary materials VI, a quantified overview can be found).

### MEAs to address clinical effectiveness uncertainties

To mitigate uncertainties around the population, outcome-based models were described in literature as potential strategies. Specifically, coverage with evidence development (CED) was proposed to gather more data on patient groups that differ from those included in the original clinical trials. It was also suggested as a form of pay-for-outcome (with rebates), linking reimbursement and payment levels to specific treatment outcomes. Mitigation strategies to address uncertainties related to clinical effectivenesswere described in diverse ways. To mitigate the uncertainty in determining the precision of the effect size, literature, on the one hand, described discounts and price-volume agreements to address uncertainties if additional therapy is needed to optimize treatment outcomes [41]. On the other hand, also more outcome-related mitigation strategies were described, such as CED and pay-for outcome. Moreover, payment at outcome achieved or different combined models, such as pay-for outcome with annuity payments could potentially be suitable [9, 49–51].

To mitigate time-related uncertainties, e.g. around the durability of the effect, both financial-based as outcome-based reimbursement models can be seen as potential mitigation strategies. Concerning payment models, only the model payment at outcome achieved was described where payments are made only after certain results have been achieved. Also, combined models can potentially be suitable, for example, when the pay-for-outcome reimbursement model is combined with annuity payments. This combination enables to capture the value of an OMP through annuity payments received over a specified period based on evidence that the treatment continues to be effective whilst also mitigating the financial risk of high upfront payments [1, 17, 24, 42–52]. Uncertainties related to the study design can potentially be mitigated through CED, payments outcomes achievement or through outcome-based combined models [4, 41, 51, 60].

### MEAs to address cost-effectiveness uncertainties

To mitigate cost-effectiveness uncertainties, strategies were mainly reported to address uncertainties around the effect and utility input parameters, as well as uncertainties related to cost-effectiveness results (e.g., incremental cost-effectiveness ratio (ICER)). These strategies included approaches such as pay-for-outcome with rebates to resolve or reduce key decision-relevant uncertainties regarding added benefit, optimal treatment schemes, and patient outcomes in order to better demonstrate value for money [53, 54]. Moreover, CED was identified as a potential mitigation strategy to address uncertainties surrounding (cost-)effectiveness. By collecting additional data, CED enables the generation of evidence on appropriate drug use or the validation of cost-effectiveness models, thereby informing future reimbursement decisions [55]. Additionally, several combined models were reported as potential strategies to mitigate uncertainties in the cost-effective conclusions, (e.g. with regard to the calculated ICER). For example, a pay-for-outcome model can be combined with rebates to link reimbursement to specific outcomes, where predetermined rebate amounts are applied if certain predefined outcomes are not achieved [15, 53].

### MEAs to address financial risks

To mitigate the high budget impact, e.g. associated with the financial risk of an unexpectedly larger number of eligible patients, price-volume agreements were frequently suggested due to the decrease in payment per patient as the number of patients or volume sold increases. Budget threshold agreements (also known as a budget cap) were also recommended. A spending limit is then set to mitigate the risk of unexpectedly high budget impact, for example when there is uncertainty on the size of the patient population [56]. Furthermore, providing free doses can ensure affordable patient access while also incentivising participation in clinical trials (e.g. for specific subpopulations with large clinical uncertainties). Here, a reduction in drug costs should improve cost-effectiveness results [57, 58]. Outcome-based combined models were reported for mitigating financial risks. For example, Wenzl et al. (2019) described how upfront payments can be combined with partial or complete refunds for non-responders or that payment can only be received for responders after the response has been established [9]. The primary purpose of these agreements is financial – to manage budget impact and reduce the average price by not paying for non-responders - rather than to reduce uncertainty around product performance [9].

Finally, the financial risk of non-appropriate use by reimbursing the treatment for patients that do not benefit most can be mitigated by ensuring only eligible patients receive treatment. Several outcome-based reimbursement models can be suitable to implement. For example, conditional treatment continuation can be seen as a mitigation strategy, as it only continues the treatment and reimburses patients who benefit from the treatment [51, 65].

**Table 1:**
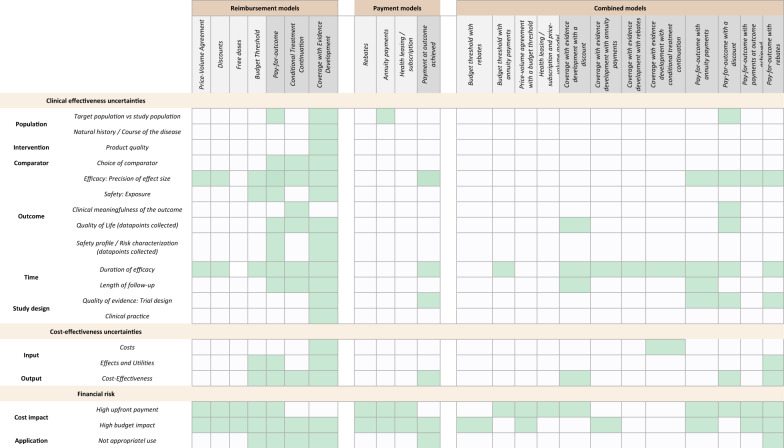
Matrix of MEA elements designed to manage reimbursement challenges for omps

*The left side of the table lists the different reimbursement challenges for OMPs as described in the literature. The top of the table shows the various types of MEAs. MEAs coloured light grey indicate financial-based models, MEAs coloured dark grey indicate outcome-based models. Light green highlights that a specific clinical-, cost-effectiveness uncertainty or/and financial risk was described as a driver for a specific MEA in literature

### Illustrating the use of the matrix: the case study of Myozyme^®^ (alglucosidase alfa)

Myozyme^®^ (alglucosidase alfa) is a therapy developed to treat patients with Pompe disease, a rare inherited disorder. It was indicated for children with early-onset Pompe disease and both adults and children with late-onset Pompe disease [59]. Table 2 shows the described reimbursement challenges for Myozyme^®^ and described MEAs as reported in the extracted literature, covering both clinical- and cost-effectiveness uncertainties and a financial risk. For example, in the article of Pouwels et al., difficulties were described in interpreting the precision of the effect size due to uncertainties about the results of the walking test and what improvements could be considered as improvements [60]. To mitigate these challenges, two different types of MEAs were described, i.e. CED and pay-for outcome with payments at outcome achieved. The matrix shows that even though CED covers the clinical- and cost-effectiveness uncertainties, the financial risk of high upfront payments is not mitigated. Implementing a pay-for outcome with payments at outcome achieved model would only be designed to mitigate one of the clinical-effectiveness uncertainties surrounding the precision of the effect size and the financial risk of high upfront payments. With the use of the developed matrix, several additional MEAs can be identified to have the opportunity to mitigate many of the reported reimbursement challenges, such as pay-for-outcome with a discount.

**Table 2:**
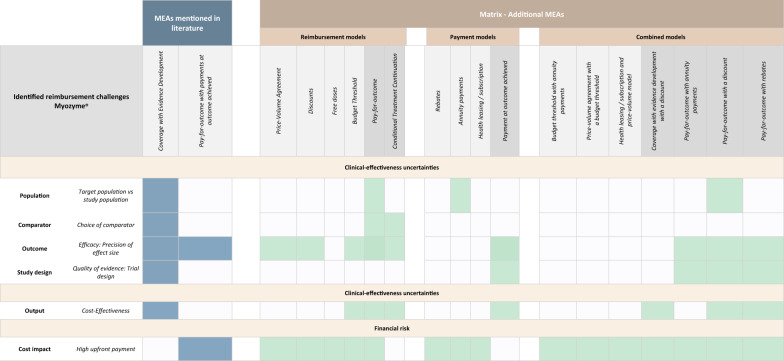
Illustrating the use of the matrix: the case study of Myozyme^®^ (alglucosidase alfa)

## Discussion

This study aimed to provide relevant stakeholders with more insight into the drivers of MEAs to reduce risk and reimbursement challenges specific to OMPs based on what was described in the literature. The matrix offers a more profound understanding of how specific MEAs can be designed to overcome reimbursement challenges specific to OMPs and mitigate clinical and cost-effectiveness uncertainties and financial risks. The results indicated that not only more commonly known schemes, such as discounts, have the potential to mitigate reimbursement challenges, but also more innovative MEAs, such as pay-for-outcome with annuity payments, and combinations thereof have been frequently described. The selected case study of Myozyme^®^ illustrated how the matrix can present stakeholders with additional suggestions to mitigate relevant reimbursement challenges.

Previous literature has outlined that even though financial-based reimbursement models are perceived as simpler and easier to implement, they can mitigate some types of uncertainty (e.g., around the size of the treatment population to limit budget impact) but do not de-risk uncertainties about the OMPs clinical effectiveness (e.g. size or persistence of effect) (Eichler, 2023). Therefore, the current understanding is often that outcome-based reimbursement models are more suitable to mitigate clinical uncertainties. Nonetheless, the matrix illustrates that others described financial-based MEAs as more suitable to mitigate uncertainties surrounding e.g., the effect size’s precision and the efficacy’s duration than financial-based models. For example, Goodman et al. described that when price-volume agreements are made on an individual level, it has the potential to mitigate the uncertainty around the precision of the effect size, e.g. when a patient needs additional therapy to improve the effectiveness of the therapy. Price-volume agreements are typically described to be applied when patient outcomes are uniform or predictable, but the anticipated number of treated patients is large or unpredictable, posing a budgetary risk [41, 50, 61–65]. These models do not entail the administrative burden of outcomes data collection or performance adjudication required for outcomes-based models [41].

Considering the outcome-based reimbursement models, many of the clinical uncertainties have the potential to be mitigated by implementing CED and pay-for-outcome models. The matrix proposes a combination of several lesser-known outcome-based models to address these uncertainties. Over the years, literature has mainly described reimbursement and payment models as mutually exclusive [9, 10, 12, 15, 17]. Our study illustrates that reimbursement and payment models can be combined; reimbursement models themselves can also be combined with each other, and multiple combinations have been reported.

### Recommendations to stakeholders

**Fig. 1:**
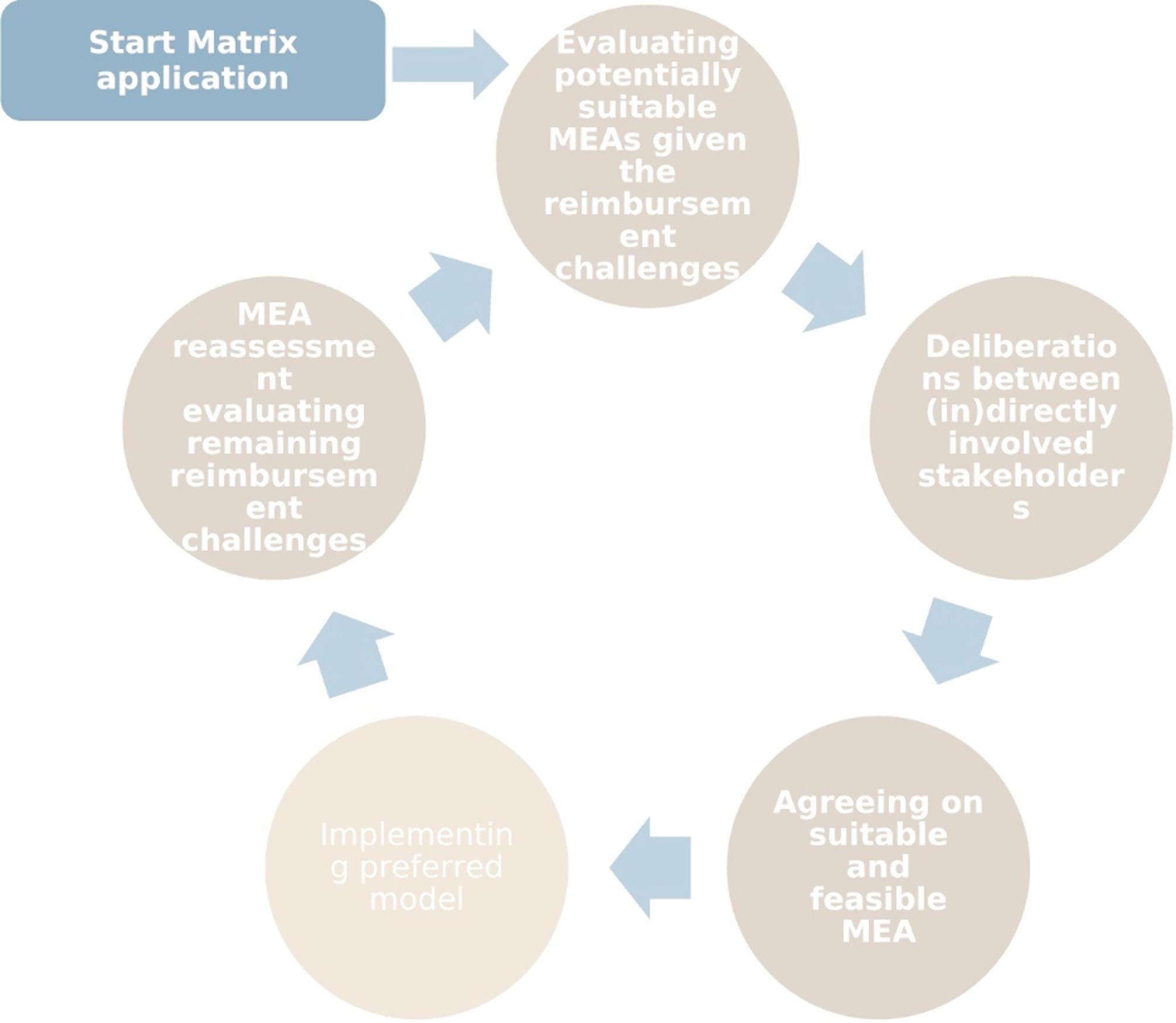
The lifecycle of a managed entry agreement and points in which the matrix can be used

To enhance the usability of the developed matrix throughout the lifecycle of an MEA for OMPs, we propose to incorporate the matrix at several points in the Fig. 1 presented below.

The developed matrix and experiences from the case study illustrate that different uncertainties and risks can require different approaches. Therefore, the evaluation of which MEA can be suitable should be started as early as possible. Preferably even before the drug approval trajectory to allow for early dialogues on which reimbursement challenges might occur and still can be overcome but also to manage expectations on what inevitable challenges will likely provide a reimbursement hurdle to start MEA preparations early on.

Through horizon scanning, HTA organisations and NCAPR can be made aware of which OMPs are in the pipeline. Early scientific/joint scientific consultation at the level of the EMA could provide HTA organisations and NCAPR with the opportunity to engage early on in the life-cycle of a new OMP to enable early identification of which reimbursement challenges will arise in terms of both clinical-, cost-effectiveness and financial risks. By making use of the developed matrix, early preparation of MEAs could be incentivised through setting up registries and aligning post-authorisation safety and/or efficacy studies imposed by regulators to be prospectively planned and aligned with post-licensing evidence generation foreseen by payers under an MEA scheme [66].

Second, research has pointed out that the perception of uncertainties and financial risks can differ between healthcare systems and stakeholders [29, 30, 67]. Therefore, we would recommend that on a national level, deliberation of (in)directly involved stakeholders in the process of reimbursement decision-making (e.g. HTDs, NCAPRs, HTA organisations, healthcare providers, patient organisations, and registry experts) should take place to gain their understanding of which challenges are most pressing to the reimbursement decision. As outlined in the matrix and showcased by the case study, a combination of multiple models can offer the potential to mitigate multiple reimbursement challenges within one strategy. The identification of the most pressing challenges could guide further determination of which MEA is most suitable given the circumstances of the OMP under assessment taken within the context of national process and stakeholder preferences.

Hereafter, the next step will be to agree on which MEA is both suitable and feasible to implement. As outlined by the case study, more complex outcome-based reimbursement and delayed payment models can be used as mitigation strategies, but so can the more simple financial-based models. HTDs and NCAPRs are advised to assess which combination of multiple MEAs/models have the feasibility to be successfully implemented and can create a compelling deal for all. If the identified MEA is not feasible—for instance, if data to address uncertainties such as generalizability cannot be collected in a reasonable timeframe or would overly burden patients and clinicians—the developed matrix can guide the exploration of alternative mitigation strategies. These may include refining the target population, implementing conditional reimbursement, or enhancing post-launch monitoring using existing data sources [6].

After agreeing on a suitable and feasible MEA, the preferred model needs to be implemented. In this phase, there is no direct need to apply the matrix. However, in the final phase, at the end of the MEA timeframe, a reassessment can take the place of not only the OMP but also the MEA structure. In principle, a simple MEA is preferred over a complex one [68, 69]. Therefore, stakeholders are advised to identify which reimbursement challenges are still prominent. If reimbursement challenges are still in need of mitigation with the use of the matrix, suitable MEAs can be evaluated and (potentially) re-entering the lifecycle of a new (simpler) MEA.

### Limitations

This study has several strengths and limitations worth noting. First, not all papers could be retrieved in the systematic literature review, which was mainly due to them not having open access or being abstracts without a full publication. Thus, not all the relevant sources may have been included in this study. Equally, the scope of the review was limited to studies in English, which means that potentially relevant sources in other languages have been missed. Nonetheless, the systematic literature review considered both relevant scientific and grey literature, including a broad scope of publications. Additionally, the use of an AI tool during the title/abstract screening minimised selection bias in this study, making us confident that the included studies will cover the array of MEAs.

New or uncommon MEAs might be very suitable even though they are described less often in contrast to the more commonly known, traditional MEAs, which are described much more often over a longer period of time. Therefore, we choose to present all MEAs that were described to be designed to mitigate OMP-specific reimbursement challenges. However, more research is needed to conclude which mitigation strategies have been found to be most suitable in practice, for which more evaluations of real-life case studies of implemented MEAs are necessary.

Third, given that the matrix is purely based on what was described in literature some inconsistencies can be found. For example, a pay-for-outcome reimbursement model was not described to mitigate uncertainties in the ‘quality of evidence: trial design; however, the combined model pay-for-outcome with a discount was. One would suspect that both MEAs have the potential to mitigate this clinical uncertainty as combining it with a discount does not initiate additional information on the quality of the evidence but mainly manages the financial risk. Therefore, some caution should be taken when interpreting the results.

Finally, for the selected case study, we only included reimbursement challenges as described in the extracted literature. However, published HTA reports highlight additional challenges. For example, in the Netherlands, the issues for Myozyme involved around the specific subgroups of the acute pediatric population versus the chronic elderly population. While the treatment effect was substantial for early-treated pediatric patients, it was, on average, relatively marginal for adults, resulting in an extremely high incremental cost-effectiveness ratio (ICER) [70]. Despite this, significant public pressure led to reimbursement for all indications. Given these challenges, other additional MEAs could be designed to mitigate these challenges.

### Future research

In the future, the scope of the research should be broadened with a wider range of therapeutic areas outside of OMPs. The developed matrix may already be used for many ATMPs, given that these therapies usually address high unmet medical needs and have an OMP status. The current matrix does not reflect feasibility considerations; therefore, to guide stakeholders on feasible MEAs, future research should focus on developing a clear outline of what practical implementation considerations should be taken into account for each MEA to enhance the more frequent and successful implementation of the preferred models and evaluate whether they did what they were supposed to do. Finally, conducting a stakeholder analysis would help verify the matrix’s validity and provide context for what MEA is suitable (and feasible) under which conditions. As previous research has outlined that different perceptions of uncertainties exist, this study could involve conducting a Delphi panel to gather valuable expert opinions until a consensus is reached. Moreover, creating more transparency in which uncertainties were perceived to be most pressing and what the main principles of the implemented MEA were, without disclosing any financial terms, would help the evaluation of what suitable and feasible mitigation strategies can be. Real-world evaluations of the proposed matrix to validate its effectiveness across diverse healthcare systems would be a useful starting point. This would ensure that the framework aligns with the needs and perspectives of key reimbursement decision-makers.

## Conclusions

The matrix provides a systematic approach applied to mitigate clinical and cost-effectiveness uncertainties and/or reimbursement challenges specific to OMPs through advising MEAs. This study highlights the diversity in the drivers of MEAs to reduce risk and reimbursement challenges specific to OMPs and underscores the relevance of considering both established and innovative MEAs to address these. It emphasises the potential of combining reimbursement and payment models to effectively address multifaceted reimbursement challenges. The matrix could provide national competent authorities for pricing and reimbursement decision-making with a stronger hold on advising MEAs. With this, it is hoped that the reimbursement scene in the scope of OMPs will be improved, where MEAs are more suited to manage the stated problems and ultimately improve patient access to OMPs.

## Supplementary Information

Supplementary Material 1

## Data Availability

The datasets during and/or analysed during the current study available from the corresponding author on reasonable request.
