# Supplementary material for: Drivers of managed entry agreements to reduce reimbursement challenges of orphan medicinal products: the development of a matrix
Source: Orphanet J Rare Dis. 2025 Oct 27;20:540. doi: 10.1186/s13023-025-04020-8 (PMC12560585; doi:10.1186/s13023-025-04020-8)
Supplement: Supplementary file 1 — Supplementary Material 1 [file 13023_2025_4020_MOESM1_ESM.docx]

### Supplementary materials I

PubMed Search String

((“2000”[Date – Publication] : “3000”[Date – Publication])) AND ((((Rare Disease OR Orphan Disease[MeSH Terms]) OR (rare disease*[Title/Abstract] OR orphan drug*[Title/Abstract] OR genetic disease*[Title/Abstract] OR rare cancer*[Title/Abstract]))) AND (((“Risk Sharing, Financial”[Mesh]) OR (managed entry agreement*[Title/Abstract] OR risk sharing agreement*[Title/Abstract] OR outcome-based agreement*[Title/Abstract] OR patient access scheme*[Title/Abstract] OR performance-based risk sharing[Title/Abstract] OR financial based agreement*[Title/Abstract] OR budget threshold[Title/Abstract] OR payment after outcome achieved[Title/Abstract] OR upfront payment*[Title/Abstract] OR subscription*[Title/Abstract] OR conditional treatment continuation[Title/Abstract] OR price-volume agreement*[Title/Abstract] OR discount*[Title/Abstract] OR rebate*[Title/Abstract] OR pay for outcome[Title/Abstract] OR annuity payment*[Title/Abstract] OR coverage with evidence development[Title/Abstract] OR payment*[Title/Abstract] OR risk sharing[Title/Abstract] OR delayed payment model*[Title/Abstract] OR pay-for-performance[Title/Abstract] OR pay at outcomes achieved[Title/Abstract] OR no cure no pay[Title/Abstract] OR Netflix[Title/Abstract]))))

###

Embase Search String

(‘rare disease’/exp OR ‘orphan drug’/exp) OR (‘rare disease*’:ab,ti,kw OR ‘orphan drug*’:ab,ti,kw OR ‘genetic disease*’:ab,ti,kw OR ‘rare cancer*’:ab,ti,kw) AND (‘managed entry agreement*’:ab,ti,kw OR ‘risk sharing agreement*’:ab,ti,kw OR ‘outcome-based agreement*’:ab,ti,kw OR ‘patient access scheme*’:ab,ti,kw OR ‘performance-based risk sharing’:ab,ti,kw OR ‘financial based agreement*’:ab,ti,kw OR ‘budget threshold’:ab,ti,kw OR ‘payment after outcome achieved’:ab,ti,kw OR ‘upfront payment*’:ab,ti,kw OR ‘subscription*’:ab,ti,kw OR ‘conditional treatment continuation’:ab,ti,kw OR ‘price-volume agreement*’:ab,ti,kw OR ‘discount*’:ab,ti,kw OR ‘rebate*’:ab,ti,kw OR ‘pay for outcome’:ab,ti,kw OR ‘annuity payment*’:ab,ti,kw OR ‘coverage with evidence development’:ab,ti,kw OR ‘payment*’:ab,ti,kw OR ‘risk sharing’:ab,ti,kw OR ‘delayed payment model*’:ab,ti,kw OR ‘pay-for-performance’:ab,ti,kw OR ‘pay at outcomes achieved’:ab,ti,kw OR ‘no cure no pay’:ab,ti,kw OR ‘netflix’:ab,ti,kw) AND [embase]/lim AND [2023]/py

### Supplementary materials II

Keywords Used in Title/Abstract Screening

Rare disease; rare diseases; Orphan drug; Health Technology Assessment; HTA; Managed Entry Agreement; Evidence; reimbursement; payment; price; Risk Sharing; coverage with evidence development; delayed payment models; uncertainty; OECD; discounts; rebates; price-volume agreements; budget threshold; pay for outcome; pay-for-performance; conditional treatment continuation; upfront payment; pay at outcomes achieved; annuity payments; health leasing; subscription; upfront payments; no cure no pay; Netflix; performance-based risk sharing.

### Supplementary materials III

Information about the selected case study

Myozyme is used to treat patients who have Pompe disease, a rare inherited disorder. Patients with Pompe disease do not have enough of an enzyme called alpha-glucosidase. This enzyme normally breaks down sugar stored as glycogen into glucose that can be used for energy by the body’s cells. If the enzyme is not present, glycogen builds up in certain tissues, particularly the muscles, including the heart and diaphragm (the main breathing muscle under the lungs). The progressive build-up of glycogen causes a wide range of symptoms, including an enlarged heart, breathing difficulties and muscle weakness. The disease can appear at birth (the ‘infantile-onset’ form) but also later in life (the ‘late-onset’ form).

### Supplementary materials IV

**
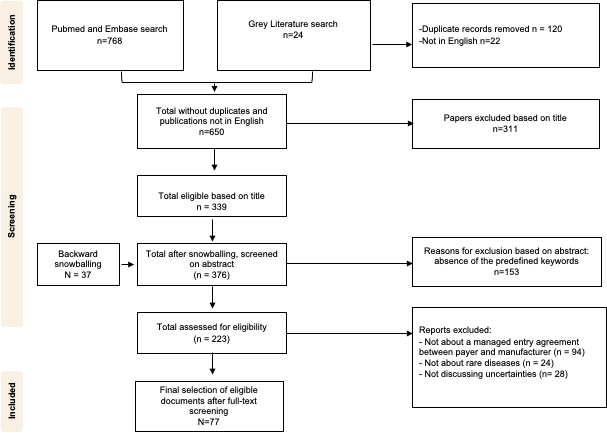
**

**Figure 1.** PRISMA diagram for the inclusion of references describing managed entry agreements and uncertainty in the context of orphan medicinal products.

### Supplementary materials V

**Box 1.** Included reimbursement and payment models

| **Financial based reimbursement models** | |
| --- | --- |
| *Discounts / rebates* | Simple price discounts, publicly or confidentially agreed upon between the payer and manufacturer. |
| *Budget threshold / dedicated funds* | The maximum amount of reimbursement for an individual innovative treatment (budget threshold) or therapeutic area (dedicated funds) to cap total expenditures. Translates into a maximum number of patients treated per year (utilization capping) or sharing of costs with the manufacturer or patients after a pre-defined budget threshold has been exceeded. |
| *Price-volume agreements* | Drug prices are progressively lowered as more patients receive the treatment. |
| **Outcome-based reimbursement models** | |
| *Value-based pricing* | Setting the price of a new medicine and/or deciding on reimbursement based on the therapeutic value that a therapy offers, usually assessed through health technology assessment (HTA). To compare value across healthcare domains incremental cost-effectiveness ratios and willingness to pay-thresholds can be used. |
| *Pay-for outcome / outcome guarantees* | The level of reimbursement is related to the future performance of the product in either research or a real world (performance-based) environment. Therapy costs are fully or partially covered by the manufacturer if outcomes are not achieved. |
| *Conditional treatment continuation* | Continuation of coverage for individual patients is conditioned upon meeting short-term treatment goals. When agreed conditions are not met, coverage will end. |
| *Coverage with evidence development* | Provisional reimbursement of promising technologies with limited clinical evidence. Temporary reimbursement is granted with an obligation for the manufacturer to obtain and provide additional data. Can be organized either with patients only having access when included in the study (only in research) or with an obligation to generate data and unrestricted access (only with research). |
| **Upfront payment** |  |
|  | Agreement to pay treatment costs upfront to the manufacturer at the time of treatment delivery. This is the most common payment model. |
| **Delayed payment models** |  |
| *Payments at outcome achieved* | Paying treatment costs only after pre-defined results have been achieved. |
| *Annuity Payments* | Spreading payments over multiple years, with an agreed upon amount of treatment or outcomes delivered. |
| *Health leasing / subscription* | Paying for unlimited use of a therapy during a predefined period. |

### Supplementary materials V

**Table 1.** Identified managed entry agreements and the categorized uncertainties they aim to mitigate.

|  | **Managed entry agreement** | **Uncertainties** | | **N** |
| --- | --- | --- | --- | --- |
| **Reimbursement models** | **Discounts/ rebates** | Clinical-effectivness uncertainty | *Efficacy: Precision of effect size* | 1 |
|  |  |  | *Duration of efficacy* | 1 |
|  |  | Financial risk | *High upfront payment* | 14 |
|  |  |  | *High budget impact* | 8 |
|  | **Price-volume agreement** | Clinical-effectivness uncertainty | *Efficacy: Precision of effect size* | 2 |
|  |  |  | *Duration of efficacy* | 2 |
|  |  | Financial risk | *High upfront payment* | 4 |
|  |  |  | *High budget impact* | 14 |
|  | **Free doses** | Financial risk | *High upfront payment* | 1 |
|  |  |  | *High budget impact* | 1 |
|  | **Budget threshold** | Clinical-effectivness uncertainty | *Efficacy: Precision of effect size* | 1 |
|  |  |  | *Safety: Exposure* | 1 |
|  |  |  | *Duration of efficacy* | 1 |
|  |  | Cost-effecttivness uncertainty | *Effects and Utilities* | 1 |
|  |  |  | *CE-results* | 1 |
|  |  | Financial risk | *High upfront payment* | 1 |
|  |  |  | *High budget impact* | 8 |
|  |  |  | *Rational use* | 1 |
|  | **Pay for outcome** | Clinical-effectivness uncertainty | *Target population vs study population* | 2 |
|  |  |  | *Choice of comparator* | 6 |
|  |  |  | *Safety: Exposure* | 1 |
|  |  |  | *Quality of Life (datapoints collected)* | 2 |
|  |  |  | *Safety profile / Risk characterization (datapoints collected)* | 1 |
|  |  |  | *Duration of efficacy* | 5 |
|  |  |  | *Length of follow-up* | 3 |
|  |  | Cost-effecttivness uncertainty | *Effects and Utilities* | 1 |
|  |  |  | *CE-results* | 3 |
|  |  | Financial risk | *High upfront payment* | 2 |
|  |  |  | *High budget impact* | 3 |
|  |  |  | *Rational use* | 1 |
|  | **Conditional treatment continuation** | Clinical-effectivness uncertainty | *Choice of comparator* | 1 |
|  |  |  | *Efficacy: Precision of effect size* | 5 |
|  |  |  | *Clinical meaningfulness of the outcome* | 1 |
|  |  |  | *Quality of Life (datapoints collected)* | 1 |
|  |  |  | *Duration of efficacy* | 2 |
|  |  |  | *Length of follow-up* | 1 |
|  |  | Cost-effecttivness uncertainty | *CE-results* | 1 |
|  |  | Financial risk | *High budget impact* | 3 |
|  |  |  | *Rational use* | 5 |
|  | **Coverage with evidence development** | Clinical-effectivness uncertainty | *Target population vs study population* | 6 |
|  |  |  | *Natural history / Course of the disease* | 4 |
|  |  |  | *Product quality* | 1 |
|  |  |  | *Choice of comparator* | 1 |
|  |  |  | *Efficacy: Precision of effect size* | 10 |
|  |  |  | *Safety: Exposure* | 1 |
|  |  |  | *Quality of Life (datapoints collected)* | 1 |
|  |  |  | *Safety profile / Risk characterization (datapoints collected)* | 1 |
|  |  |  | *Duration of efficacy* | 15 |
|  |  |  | *Length of follow-up* | 5 |
|  |  |  | *Quality of evidence: Trial design* | 5 |
|  |  |  | *Clinical practice* | 2 |
|  |  | Cost-effecttivness uncertainty | *Input parameter: Costs* | 1 |
|  |  |  | *Effects and Utilities* | 6 |
|  |  |  | *Cost-Effectiveness* | 8 |
|  |  | Financial risk | *High budget impact* | 5 |
|  |  |  | *Rational use* | 2 |
| **Payment models** | **Rebates** | Financial risk | *High budget impact* | 3 |
|  |  |  | *Rational use* | 2 |
|  | **Annuity payments** | Clinical-effectivness uncertainty | *Target population vs study population* | 1 |
|  |  | Financial risk | *High upfront payment* | 9 |
|  |  |  | *High budget impact* | 5 |
|  | **Payments at outcomes achieved** | Clinical-effectivness uncertainty | *Efficacy: Precision of effect size* | 6 |
|  |  |  | *Duration of efficacy* | 3 |
|  |  |  | *Quality of evidence: Trial design* | 1 |
|  |  | Cost-effecttivness uncertainty | *CE-results* | 1 |
|  |  | Financial risk | *High budget impact* | 2 |
|  |  |  | *Rational use* | 2 |
|  | **Health leasing and subscription** | Financial risk | *High upfront payment* | 1 |
|  |  |  | *High budget impact* | 4 |
| **Combined models** | **Budget threshold with rebates** | Financial risk | *High budget impact* | 2 |
|  | **Budget threshold with annuity payments** | Clinical-effectivness uncertainty | *Duration of efficacy* | 1 |
|  |  | Financial risk | *High upfront payment* | 1 |
|  | **Price-volume agreement with a budget threshold** | Financial risk | *High upfront payment* | 2 |
|  |  |  | *High budget impact* | 3 |
|  | **Health leasing / subscription and price-volume model** | Financial risk | *High upfront payment* | 1 |
|  | **Coverage with evidence development with a discount** | Clinical-effectivness uncertainty | *Quality of Life (datapoints collected)* | 1 |
|  |  |  | *Duration of efficacy* | 1 |
|  |  |  | *Length of follow-up* | 1 |
|  |  | Cost-effecttivness uncertainty | *CE-results* | 1 |
|  |  | Financial risk | *High upfront payment* | 3 |
|  | **Coverage with evidence development with annuity payments** | Clinical-effectivness uncertainty | *Duration of efficacy* | 2 |
|  |  | Financial risk | *High budget impact* | 1 |
|  | **Coverage with evidence development with rebates** | Clinical-effectivness uncertainty | *Duration of efficacy* | 1 |
|  | **Coverage with evidence development with conditional treatment continuation** | Clinical-effectivness uncertainty | *Duration of efficacy* | 1 |
|  |  | Cost-effecttivness uncertainty | *Input parameter: Costs* | 1 |
|  | **Pay-for-outcome with annuity payments** | Clinical-effectivness uncertainty | *Efficacy: Precision of effect size* | 6 |
|  |  |  | *Duration of efficacy* | 9 |
|  |  |  | *Length of follow-up* | 1 |
|  |  |  | *Quality of evidence: Trial design* | 3 |
|  |  | Financial risk | *High upfront payment* | 4 |
|  |  |  | *High budget impact* | 3 |
|  | **Pay-for-outcome with a discount** | Clinical-effectivness uncertainty | *Target population vs study population* | 1 |
|  |  |  | *Efficacy: Precision of effect size* | 2 |
|  |  |  | *Clinical meaningfulness of the outcome* | 1 |
|  |  |  | *Quality of Life (datapoints collected)* | 1 |
|  |  |  | *Duration of efficacy* | 1 |
|  |  |  | *Quality of evidence: Trial design* | 1 |
|  |  | Cost-effecttivness uncertainty | *CE-results* | 1 |
|  |  | Financial risk | *High upfront payment* | 1 |
|  | **Pay-for-outcome with payments at outcome achieved** | Clinical-effectivness uncertainty | *Efficacy: Precision of effect size* | 2 |
|  |  | Financial risk | *High upfront payment* | 1 |
|  |  |  | *High budget impact* | 1 |
|  | **Pay-for-outcome with rebates** | Clinical-effectivness uncertainty | *Efficacy: Precision of effect size* | 9 |
|  |  |  | *Duration of efficacy* | 4 |
|  |  |  | *Quality of evidence: Trial design* | 2 |
|  |  | Cost-effecttivness uncertainty | *Input parameters: Effects and Utilities* | 1 |
|  |  |  | *CE-results* | 2 |
|  |  | Financial risk | *High upfront payment* | 3 |
|  |  |  | *High budget impact* | 2 |
|  |  |  | *Rational use* | 1 |
